# Complete atrioventricular block as an initial manifestation of recurred oral cavity cancer: a case report

**DOI:** 10.1186/s12872-018-0876-3

**Published:** 2018-07-09

**Authors:** Jae Yeong Cho, Kye Hun Kim, Hyukjin Park, Hyun Ju Yoon, Jong Chun Park

**Affiliations:** Department of Cardiovascular Medicine, Chonnam National University Hospital, 42 Jebong-ro, Dong-gu, Gwangju, 61469 South Korea

**Keywords:** Oral cancer, Recurrence, Metastasis, Atrioventricular block

## Abstract

**Background:**

Intracardiac invasion of head and neck cancer is extremely rare. Here, we report a case of recurred oral cavity cancer presenting with complete atrioventricular (AV) block caused by cardiac metastasis.

**Case presentation:**

A 70-year-old male presented with dizziness for 2 days. He had a history of oral cavity cancer a year ago, and the tumor was treated by surgical excision after induction chemotherapy and concurrent chemoradiation therapy. Electrocardiography showed complete AV block with ventricular escape rate of 43 beats per minute. Cardiac imaging revealed about 4.0 × 2.0 cm-sized mass invading interventricular septum and AV nodes and protruding into the right ventricle. Magenetic resonance imaging of head and neck demonstrated recurred mass in oral cavity and maxillary sinus. Fluorodeoxyglucose-positron emission tomography showed hypermetabolic lesion in both oral cavity and the heart around interventricular septum and atrioventricular node indicating recurred oral cavity cancer with cardiac metastasis. Permament pacemaker of DDD type was implanted for the symptomatic complete AV block, and palliative chemotherapy was initiated.

**Conclusion:**

The present case demonstrated that oral cavity cancer can metastasize to the heart, and complete AV block may be an initial manifestation of the recurrence of extracardiac cancer with intracardiac invasion.

## Background

Head and neck cancer usually metastasize to lung, bone, and liver, but the invasion of intracardiac sturctures by head and neck cancer are extremely rare [1, 2]. Here, we report a case of recurred oral cavity cancer presenting with complete atrioventricular (AV) block caused by cardiac metastasis including AV node. We perform this case report according to the CARE guideline and its methodology.

## Case presentation

A 70-year-old male presented with dizziness for 2 days. He had a history of oral cavity cancer a year ago. Three cycles of induction chemotherapy with a combination of docetaxel 70 mg/m^2^/day, cisplatin 75 mg/m^2^/day, and 5-fluorouracil 1000 mg/m^2^/day (DCF) for 4 days at each cycle for 8 weeks and concurrent chemoradiation therapy (CCRT) with a total of 33 times of radiation (200 cGy per fraction at one time) and weekly cisplatin 30 mg/m^2^ for 8 more weeks were done. Follow-up paranasal sinus computed tomography showed decreased size of enhancing mass with necrotic change within anterior hard palate, with bony destruction of maxilla. Since only a partial response was obtained after CCRT, the tumor was treated by complete surgical excision. On histopathologic examination, the tumor was proved to be a differentiated squamous cell carcinoma. After surgical treatment, there was no definite evidence of recurrence for 6 months.

**Fig. 1:**
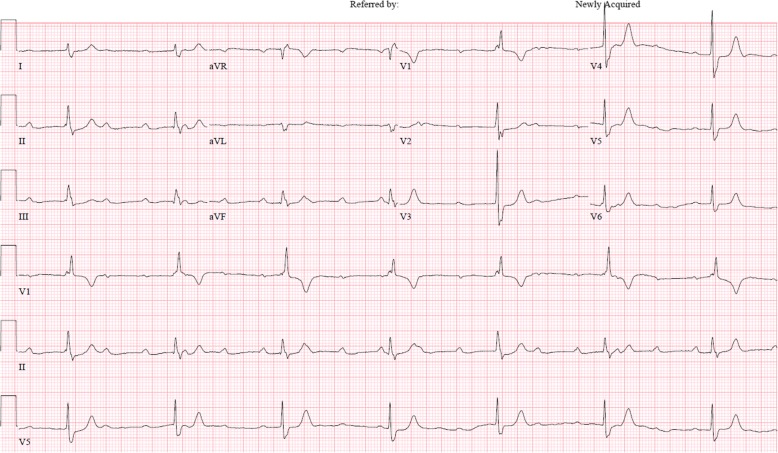
Electrocardiography showed cocmplete atrioventricular block with ventricular escape rate of 43/min

**Fig. 2:**
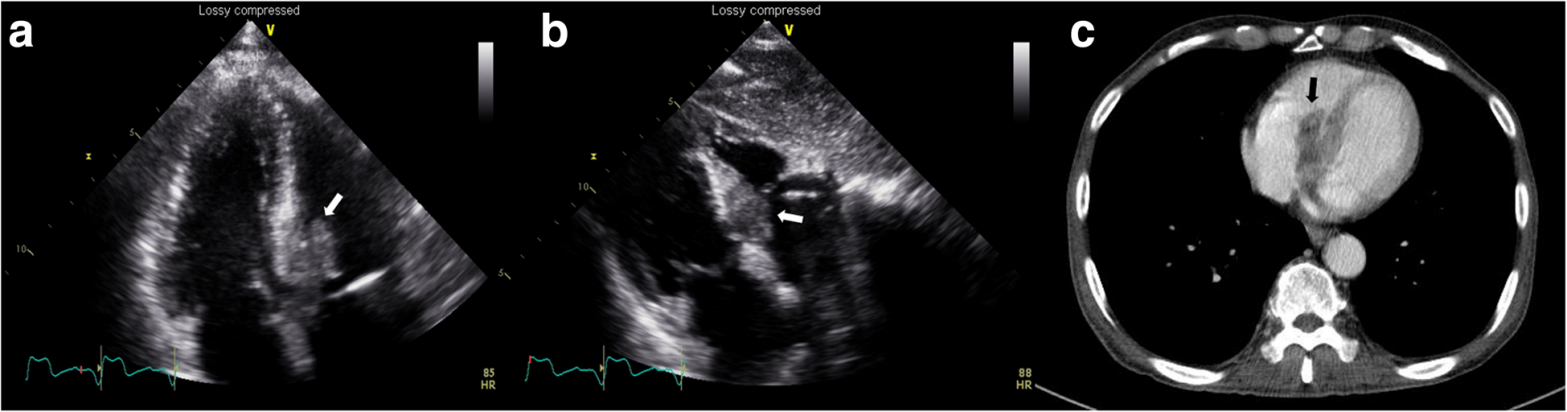
Echocardiography (**a** and **b**) and chest computed tomography (**c**) revealed about 4.0 × 2.0 cm-sized hypoechoic mass arising from the interventricular septum in the vicinity of AV node and protruding into the right ventricle

**Fig. 3:**
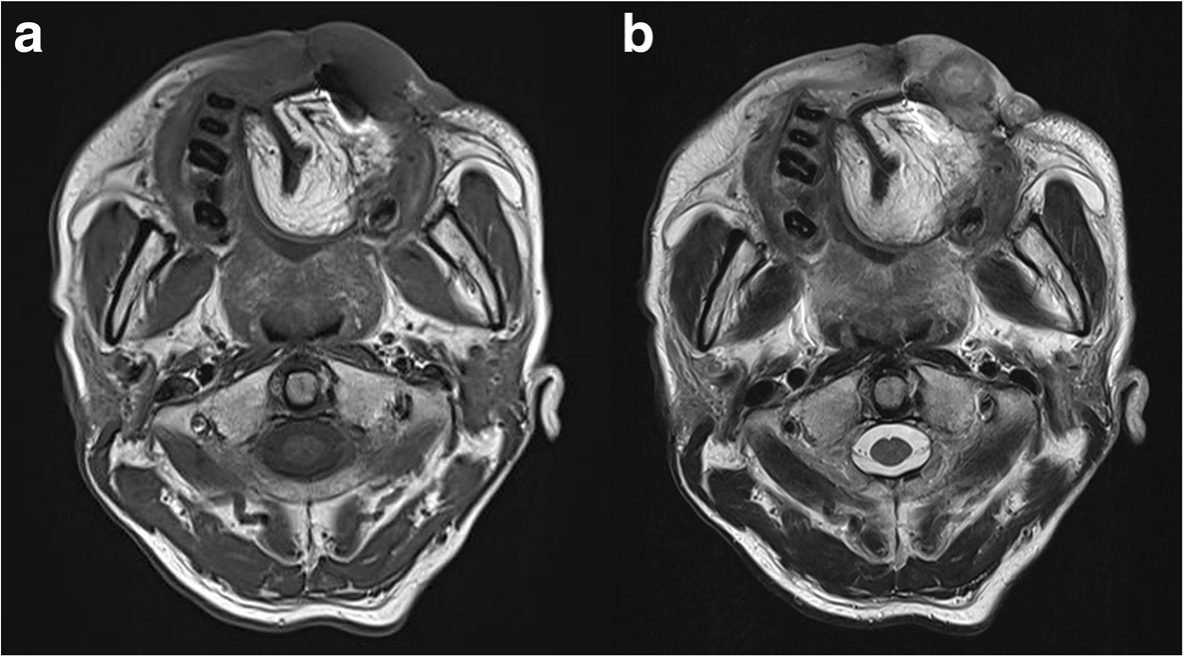
Magnetic resonance imaging of head and neck showed recurred oral cavity cancer. Lobulated soft tissue mass with bony destruction involving anterior hard palate and both anterior alveolar ridge in the T1-weighted image (**a**) and T2-weighted image (**b**) was noted

**Fig. 4:**
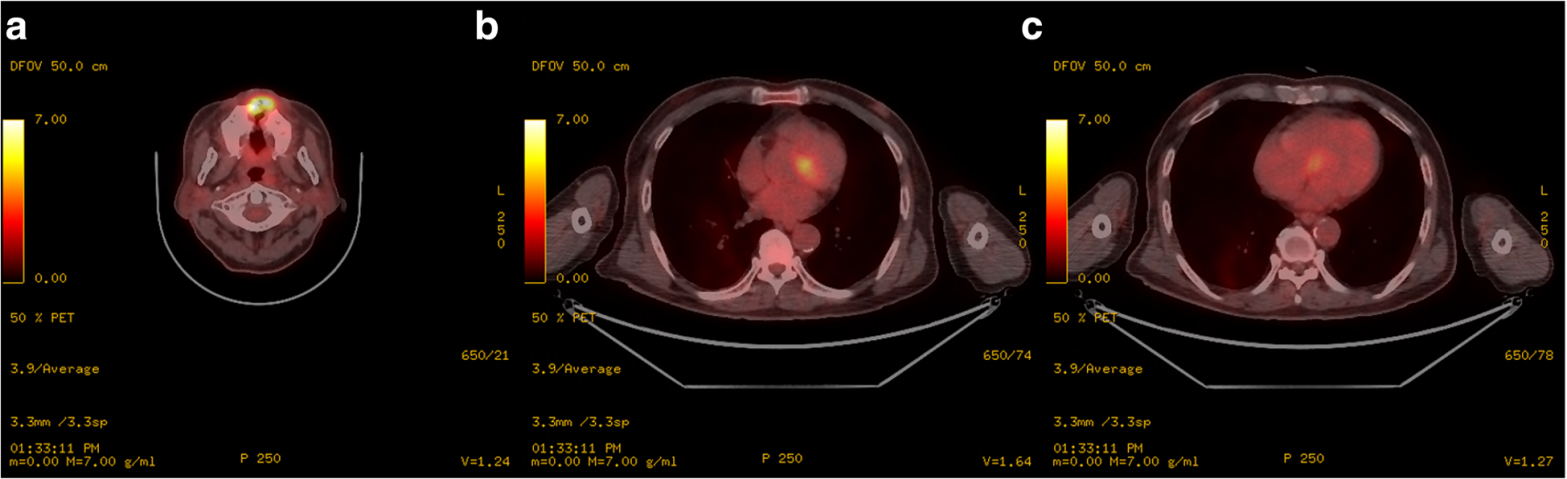
Fluorodeoxyglucose-positron emission tomography showed hypermetabolic lesion in both oral cavity (**a**) and interventricular septum of the heart (**b** and **c**)

Electrocardigraphy (ECG) at current admission showed complete AV block with a ventricular rate of 43/min (Fig. 1). Echocardiography and chest computed tomography revealed about 4.0 × 2.0 cm-sized hypoechoic mass arising from the interventricular septum in the vicinity of AV node and protruding into the right ventricle (Fig. 2). Magnetic resonance imaging of head and neck to evaluate the origin site of metastatic cardiac tumor revealed recurred mass in oral cavity and maxillary sinus (Fig. 3). Fluorodeoxyglucose-positron emission tomography (FDG-PET) showed hypermetabolic lesion in both oral cavity and the heart around interventricular septum and atrioventricular node (Fig. 4). Temporary pacemaker was inserted for complete AV block, but sinus rhythm was not restored despite 3 days of temporary pacing. A permament pacemaker of DDD type was implanted, and the patient was refered to oncology department for a palliative chemotherapy.

## Discussion and conclusions

Although any type of tumor can metastasize to the heart, intracardiac invasion of head and neck cancer is rare. In patients with oral cavity cancer, distant metastasis to other organs and distant lymph nodes is generally a late event and usually represents an incurable disease [3]. The lung is the most common site of distant spread, and bone metastasis can also occur in case of other widespread metastatic disease [4, 5]. However, distant metastasis to the heart by oral cavity cancer is extremely rare. In a study, oral cavity cancer had a invasion to the heart in 4 out of 75 cases (5.3%), while melanoma had 22 out of 79 (27.8%), adenocarcinoma of lung had 97 out of 460 (21.0%), and over all incidence were 662 out of 7289 (9.1%) [6]. Apart from the lungs, screening for distant metastases is routinely not performed in initial staging of patients with head and neck cancer [4]. However, some studies have shown that FDG-PET is valuable in detecting distant metastasis in advanced head and neck cancer, suggesting a role for whole-body FDG-PET scanning for initial staging [7–9]. FDG-PET was also useful in the present case to demonstrate the recurrence of oral cavity cancer and intracardiac metastasis. To confirm the cardiac mass is originated from the metastasis from the recurred oral cavity cancer, histopathologic examinations by tissue biopsy from these 2 different sites or tissue characterizations by both cardiac MRI and head and neck MRI would be essential. However, intracardiac biopsy was not performed because of the patient’s refusal and cardiac MRI could not be performed because the implanation of pacemaker was an obstacle for MRI. This issue would be the main limitation of this case. Considering clinical and imaging findings including FDG-PET, however, it is concluded that cardiac mass would be an metastatic lesion from the recurred oral cavity cancer.

In the present case, complete AV block on ECG was an initial clue for the recurrence of the previously treated oral cavity cancer and distant metastasis to the heart. Therefore, it is suggested that routine ECG may be incorporated into the surveillance for oral cavity cancer recurrence. In addition, further investigation is needed for the outcomes of AV block after chemotherapy for recurred cancer, which is totally unknown.

In conclusion, the present case demonstrated that oral cavity cancer can metastasize to the heart and complete AV block may be an initial manifestation of the recurrence of extracardiac cancer with intracardiac invasion. Therefore, distant metasis including cardiac metastasis should thoroughly evaluated by using multimodality imagings including echocardiography, computed tomography, magnetic resonance, and PET in patients with oral cavity cancer.

## Abbreviations

AV: Atrioventricular
ECG: Electrocardiography
FDG: Fluorodeoxyglucose
PET: Positron emission tomography
